# Long-term recurrence of nonmelanoma skin cancer after topical methylaminolevulinate photodynamic therapy in a dermato-oncology department*

**DOI:** 10.1590/abd1806-4841.20154080

**Published:** 2015

**Authors:** Joana Cabete, Margarida Rafael, Mariana Cravo, Cecília Moura, Fernanda Sachse, Manuela Pecegueiro

**Affiliations:** 1 Hospital de Santo António dos Capuchos, Centro Hospitalar de Lisboa Central - Lisbon, Portugal; 2 Instituto Português de Oncologia de Lisboa Francisco Gentil - Lisbon, Portugal

**Keywords:** Bowen's disease, Carcinoma, basal cell, Skin neoplasms

## Abstract

**BACKGROUND:**

Most available studies on the efficacy of topical photodynamic therapy focus on short-to medium-term results. Long-term data are scarce.

**OBJECTIVE:**

To evaluate the long-term efficacy of photodynamic therapy with topical methylaminolevulinate to treat Bowen's disease and basal cell carcinoma in the clinical practice setting of a dermato-oncology department.

**METHODS:**

The study included patients diagnosed with Bowen's disease or basal cell carcinoma, and who received photodynamic therapy from 2004 to 2008. Treatment protocol and clinical follow-up were standardized. The primary endpoint was clinically observed recurrence in a previous photodynamic therapy-treated area. Descriptive and survival analyses were performed.

**RESULTS:**

A total of 31 Bowen's disease lesions and 44 superficial basal cell carcinoma were treated, with a median follow-up of 43.5 months. Recurrence was observed in 14 Bowen's disease lesions (53.8%) and in 11 superficial basal cell carcinoma (33.3%). Significantly higher estimates for recurrence rates were found in patients with Bowen's disease (p=0.0036) or those aged under 58 years (p=0.039). The risk of recurrence was higher in patients with Bowen's disease than in those with superficial basal cell carcinoma and younger patients.

**CONCLUSIONS:**

Recurrence should be considered when choosing to treat non-melanoma skin cancer with photodynamic therapy. Younger age and Bowen's disease were independent predictors for long-term recurrence, suggesting the need to establish an extended period of follow-up for this subset of patients.

## INTRODUCTION

Nonmelanoma skin cancer (NMSC) is the most common cancer in white-skinned individuals and its incidence is increasing worldwide.^1^ Treatment approaches for NMSC are predominantly curative and surgery remains the mainstay of care. However, nonsurgical options have emerged as viable alternatives in patients who are either poor surgical candidates or have tumours that are not amenable to surgical treatment, namely multifocal lesions, extensive disease, lesions in cosmetically sensitive areas, or difficult locations. Topical photodynamic therapy (PDT) is a widely used, non-invasive treatment option for certain NMSCs. It is approved in Europe for the treatment of actinic keratosis, Bowen's disease (BD), superficial basal cell carcinomas (sBCC), and thin nodular basal cell carcinomas (nBCC).^2^ High efficacy is reported when using standardized protocols, along with superior cosmetic outcomes compared with other therapies.^2^ Nevertheless, long-term recurrence studies based on clinical practice are scarce.

The present study aimed to evaluate the long-term efficacy of PDT with topical methylaminolevulinate (MAL) in treating BD and BCC in a dermato-oncology department, within a normal context of medical practice.

## PATIENTS AND METHODS

### Patients

A retrospective cohort study was conducted in a tertiary dermatology department at an oncology hospital. All adult patients diagnosed with BD or BCC and treated inclusively with methylaminolevulinate photodynamic therapy (MAL-PDT) from 2004 to 2008, were enrolled. The Ethics Committee approved the study protocol.

### Treatment and evaluation protocol

MAL-PDT was delivered using a standardized protocol of two treatments one week apart, repeated at 3 months in cases of tumour persistence. Lesion preparation was performed before each treatment according to tumour thickness and hyperkeratosis. Gentle curettage was used for debridement of superficial lesions, while debulking curettage was preferred for nodular tumours. Approximately 1mm-thick methylaminolevulinate 160mg/g cream (Metvix^®^; Galderma International, Paris, France) was applied, covering the entire lesion and extending 5mm beyond the clinical tumour margins. An occlusive, adhesive dressing was then applied for at least 3 hours, after which the cream was removed with a 0.9% saline solution. Fluorescence was visualized with a Wood's light before treatment. The lesion area was illuminated with a red (630nm wavelength) light source (Aktilite^®^; Photocure ASA, Oslo, Norway), delivering a total dose of 3740 J/cm^2^ (depending on the guidelines at the time of treatment) during an exposure time of 8'20".

Clinical follow-up was performed 1, 3, and 6 months following treatment and thereafter every 6 months. The primary endpoint was clinically observed recurrence in a previous PDT-treated area. Treated areas were identified with the help of standard illustrations on which the lesions had been signed and clinical pictures. Recurrence was treated at the discretion of the clinician. Clinical records were retrospectively reviewed, and data regarding demographic and tumour characteristics, along with treatment outcomes and recurrence, were registered.

### Statistical analysis

Descriptive statistics are presented as a percentage for categorical variables, and as a mean with standard deviation or median with interquartile range for continuous variables, after testing for normality with the Kolmogorov-Smirnov test.

Variables considered for survival analysis included: patient's age, gender, diagnosis, tumour location and tumour size. Survival analysis was performed with the Kaplan-Meier method, followed by nonparametric comparison of subgroups. Multivariate survival analysis was conducted by applying the Cox regression model to factors found to be predictive for recurrence on univariate analysis. Hazard-ratios (HR) were estimated using a 95% confidence interval (CI). The level of statistical significance was set at α=0.05. Statistical analysis was carried out using the Statistical Package for the Social Sciences (SPSS) version 21 (SPSS, Chicago, IL).

## RESULTS

### Descriptive analysis

MAL-PDT was used to treat a total of 77 tumours (corresponding to 67 patients). Of these, 54.5% were diagnosed in female patients and 45.5% in male patients, with a median age of 71 (P_25_;P_75_=30;92). BD was diagnosed in 31 tumours (40.3%), sBCC in 44 (57.1%), and nBCC in 2 (2.6%). A diagnostic biopsy was performed in 53 (68.8%) lesions. There was a history of other malignant or pre-malignant lesions in 62.3% of the cases treated. The median follow-up was of 43.5 months (P_25_;P_75_=3;100). The baseline characteristics of tumours are shown in table 1.

**Table 1:** Baseline characteristics of the tumours (n=77)

Gender, n (%)	
Male	35 (45.5)
Female	42 (54.5)
Age, median (P25;P75), years	71 (30;92)
Diagnosis, n (%)	
Bowen's disease	31 (40.3)
Superficial basal cell carcinoma	44 (57.1)
Nodular basal cell carcinoma	2 (2.6)
Biopsy before treatment, n (%)	
Bowen's disease	23 (74.2)
Superficial basal cell carcinoma	30 (68.2)
Location of lesions, n (%)	
Head	26 (33.8)
Trunk	28 (36.3)
Limbs	23 (29.9)
Tumour area, median (P25;P75), cm2	5 (0.25; 100)
Other (pre)malignant lesions, n (%)	48 (62.3)
Follow-up, median (P25;P75), months	
Overall	43.5 (3; 100)
Bowen's disease	42 (3; 99)
Superficial basal cell carcinoma	49 (3; 100)

As only 2 nBCCs were submitted to treatment, and since neither responded to debulking followed by PDT, they were excluded from further analysis.

Three months after the last PDT treatment, complete response was observed in 26 BD lesions (83.9%) and in 33 sBCCs (75%) (Table 2). Recurrence was observed in 25 of these 77 tumours: 14 involving BD (53.8%) and 11 sBCCs (33.3%). Similar recurrence rates were found in the subgroup of previously biopsied tumours (55.6% in BD and 31.8% in sBCC). Eight (57.1%) BD and one (9.1%) sBCC recurrences were confirmed by histopathological examination.

**Table 2:** Treatment outcomes 3 months after treatment and long-term recurrence after complete response

	Bowen's disease	Superficial basal cell carcinoma	p-value
Complete Response, n (%)	26/31 (83.9)	33/44 (75)	0.405
Partial Response, n (%)	4/31 (12.9)	9/44 (20.5)	0.539
No Response, n (%)	1/31 (3.2)	2/44 (4.5)	1
Recurrence, n (%)	14/26 (53.8)	11/33 (33.3)	0.085

### Survival analysis

When tumours were analyzed using a time-toevent approach, diagnosis and age subgroups exhibited statistically significant differences in recurrence rates. Moreover, diagnosis and age were found to be independent predictors for clinical recurrence in multivariate Cox analysis. Other factors, such as gender, tumour location and tumour size, did not have a statistically significant impact on recurrence in univariate analysis and therefore were not considered for the final regression model.

**Figure 1:**
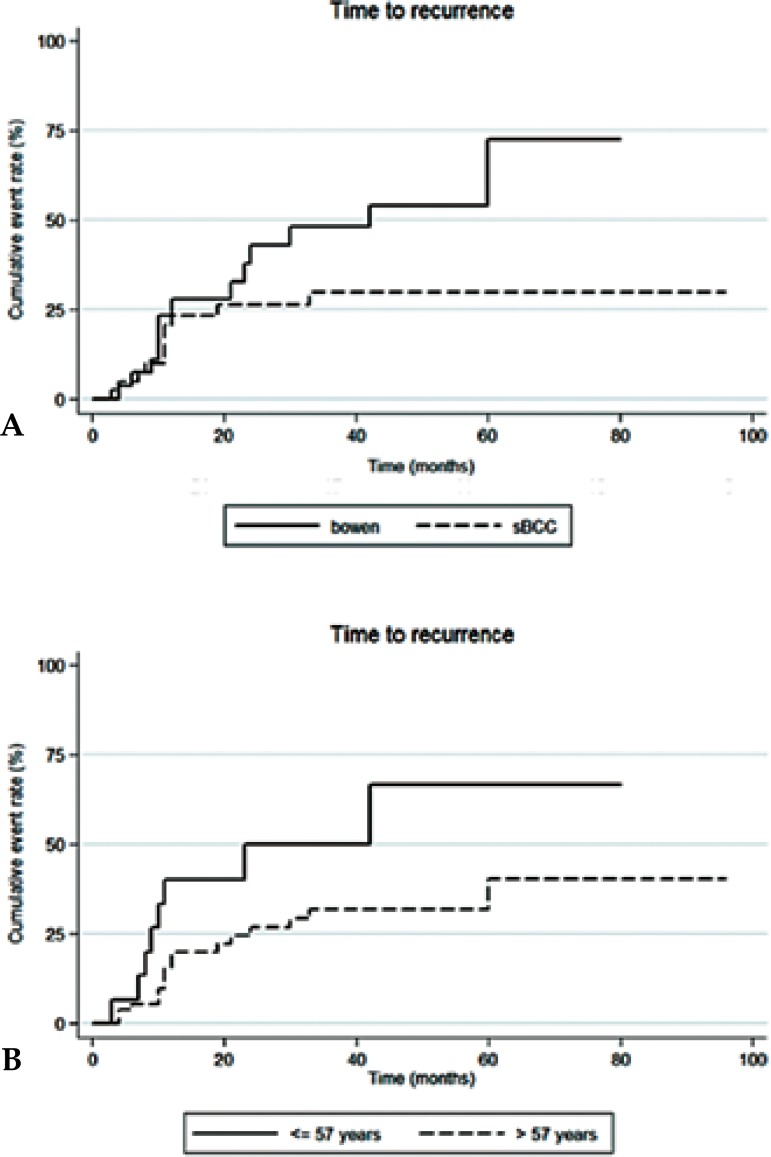
**A)** Estimated recurrence rates by diagnosis; **B)** Estimated recurrence rates by age group

• Diagnosis and recurrence:

Lesion recurrence during follow-up was significantly higher for BD than for sBCC (p=0.004). The estimated recurrence rates for BD were 7.4% at 6 months, 27.9% at 12 months, 43% at 24 months, 48.2% at 36 months, 54% at 48 months, and 72.4% at 60 months. Estimated recurrence rates for sBCC were 5% at 6 months, 23.4% at 12 months, 26.5% at 24 months, and 30% from 36 months onward. This concludes a 5-year estimated recurrence rate of 72.4% in BD vs. 30% in sBCC (Figure 1A).

Patients with BD had a significantly greater risk of recurrence than those with sBCC (HR 2.38, 95% CI: 1.07-5.59, p=0.033).

• Age and recurrence:

An optimal cutpoint for the continuous covariate "age" was calculated using martingale residuals, and set at 57 years. Patients aged 57 or younger experienced higher tumour recurrence in comparison with older patients through follow-up time (p=0.039). The estimated 5-year recurrence rates were 66.7% for the former vs. 40.4% for the latter (Figure 1B). Specifically, the estimated recurrence rates for patients aged 57 or under were 6.7% at 6 months, 40% at 12 months, 50% at 24 and 36 months, and 66.7% from 48 months onward. Patients aged over 57 had estimated recurrence rates of 5.6% at 6 months, 20% at 12 months, 26.9% at 24 months, 31.9% at 36 and 48 months, and 40.4% at 60 months.

The risk of recurrence was significantly higher in patients aged 57 or under than in those aged above 57 (HR 2.76, 95% CI: 1.17-6.51, p=0.02).

Differences in recurrence rates according to diagnosis were statistically insignificant in both age subgroups.

## DISCUSSION

Current guidelines propose PDT as an effective treatment for NMSC.^2,3^ Efficacy, together with good tolerability, a tissue-sparing attitude, easy treatment protocols, good wound-healing and favourable cosmesis, make it an appealing treatment option for both doctors and patients. While widely accepted and increasingly used, most available studies focus on short-to medium-term results regarding both efficacy and cosmesis. Long-term data are still scarce.

Despite the many available options for BD management, there is no gold standard treatment for this intra-epidermal squamous cell carcinoma (SCC).^2,3^ However, PDT has merited a strength of recommendation A and a level of evidence 1, in the light of presently available studies.^2,3^ Complete response rates of 88-100% were reported 3 months after MALPDT treatment for BD, thus justifying this rating.^3^ The majority of long-term studies report to follow-up periods of 12-24 months for 23-103 BD lesions: two studies presented recurrence rates of 13.6% and 14.6% at 12 months; 13% of the lesions recurred in a 16.6-month study; and a 24-month follow-up analysis reported a 29.3% recurrence rate.^4-7^ One report had a longer follow-up period of 50 months, with a recurrence of 11.6% in 43 of the lesions studied, all occurring in the first year after treatment.^8^ These figures contrast with those obtained in the present study. Briefly, the estimated recurrence rates for BD were 27.9% at 12 months, 43% at 24 months, 54% at 48 months, and 72.4% at 60 months. Whereas these data are a product of a time-to-event approach through survival analysis, the overall recurrence of 53.8% (55.6% in biopsied lesions), is also consistently high. The main explanation may reside in the particular characteristics of the population studied. As a tertiary care dermatology unit within an oncology hospital, most referrals comprise complex cases, patients with many or extensive lesions in difficult-to-treat areas, occasionally with ill-defined borders. Field carcinogenesis, a common background in the actinic damaged skin of patients with SCC, may also account for the high recurrence rates in these patients, especially when PDT is used as a lesion-directed therapy.

Superficial therapies have been explored for the treatment of sBCC. Their increasing use in clinical practice is supported by the recognition of sBCC as a low-risk, slow-growing epidermal tumour. Similarly to BD, PDT is recommended in European guidelines with strength of recommendation A and level of evidence 1.^2^ Treatment of sBCC with MAL-PDT is highly effective, with most studies reporting short-term efficacy rates of over 90% and good to excellent cosmetic results.^2^ Follow-up research on MAL-PDT is outlined in a few studies.^9^ Recurrence rates of 9.3% and 13% were reported in two 12-month follow-up evaluations.^10,11^ A 22% recurrence was found at 24 months in one study, while in another, a time-to-event approach applied to the same period estimated a lesion clearance rate of 82% for sBCC.^12,13^ Finally, two studies reporting to longer follow-up assessments determined a recurrence rate of 22% at 48 and 60 months.^14,15^ These literature findings generally agree with the results herein presented: an overall recurrence of 33.3% (31.8% in biopsied lesions) for a median follow-up period of 49 months, and estimated recurrence rates of 23.4% at 12 months, 26.5% at 24 months, and 30% at 60 months.

Superficial BCC tends to have a less aggressive biological behaviour than BD. The latter carries a risk of invasive carcinoma of about 3-5%.^16^ This may explain the significantly higher recurrence rates of BD compared with sBCC found in this study.

Young age is certainly not a consensual risk factor in biomedical literature on NMSC.^17-19^ Most guidelines do not consider it a high-risk factor for recurrence or aggressive behaviour. Nevertheless, age appears as a predictor for recurrence in the present study, with an almost three-fold increased risk of recurrence in patients aged 57 years or younger. It is possible that, once again, population characteristics have contributed to this finding. They include likely actinic damage sustained at an earlier age, suggested by the history of other premalignant and malignant skin lesions. There may also be a difference in response to PDT between young and aged skin. Differences in skin thickness may be attributable to a modified penetration of both the photosensitizer and light. Nevertheless, no published data refer to such a discrepancy.

We acknowledge some limitations to this study. As a retrospective study, the amount of collectable data is limited. For instance, it would have been fruitful to detail personal history so as to include immunosuppression or exposure to radiotherapy. Another limitation is the non-consistent execution of diagnostic biopsies in the primary and recurrent lesions. However, this study was undertaken in a setting of daily clinical practice. As a non-invasive treatment, dermatologists may instigate PDT without first resorting to a biopsy. In routine clinical practice, the diagnosis is made on clinical grounds, usually with the aid of dermoscopy. Doubtful, ulcerated, or pigmented lesions, those occurring in immunosuppressed patients and recurrences, should be systematically biopsied.^20^ In this study, overall recurrence rates were similar to the recurrence rates of biopsy-proven lesions, both for the BD and sBCC groups. Nevertheless, given the higher recurrence rates of BD in the present study, none of the recurrences sent for histopathology was diagnosed as invasive SCC.

## CONCLUSIONS

This study suggests that recurrence is frequent and should be taken into account when choosing to treat NMSC with MAL-PDT. The risk of recurrence was significantly higher in patients with BD or younger patients, highlighting the particularly low long-term efficacy of MAL-PDT in treating BD among the population studied. Although this reflects the reality of one institution, it stresses the need to establish an extended period of follow-up for patients with BD. While surgery should be considered, we believe that PDT can be used for BD because recurrence usually occurs in smaller foci, easily managed by PDT or other treatment modalities. Field-directed therapy may also be a better option for patients with actinic damage. Finally, efficacy, long-term recurrence, and cosmetic outcomes should be weighted to patients' characteris tics (including diagnosis and age), and the therapeutic choice should be made on a case-by-case basis.
